# Case Report: A case of hypertrophic lupus erythematosus with negative CD123 staining and absence of transepidermal elimination of elastin

**DOI:** 10.12688/f1000research.3267.2

**Published:** 2014-06-04

**Authors:** Matthew Hughes, Jerad M. Gardner, Ling Gao

**Affiliations:** 1 Department of Dermatology, University of Arkansas for Medical Sciences, Little Rock, AR 72205, USA; 2 Department of Pathology, University of Arkansas for Medical Sciences, Little Rock, AR 72205, USA

## Abstract

We report the case of a 49-year-old male with clinical and histological findings consistent with hypertrophic lupus erythematosus (HLE). HLE must be clinically and histologically differentiated from keratoacanthoma, hypertrophic lichen planus, squamous cell carcinoma and plaque type psoriasis. CD123 positivity and transepidermal elimination of elastin have recently been reported as tools to distinguish HLE. Interestingly, in this case, biopsies of two separate lesions failed to reveal these two features. The etiology of this discrepancy is unknown and further studies are needed to clarify the utility of CD123 positivity and transepidermal elimination of elastin in the diagnosis of hypertrophic lupus erythematosus.

## Introduction

Hypertrophic lupus erythematosus (HLE) is a rare subset of discoid lupus erythematosus, characterized by erythematous, indurated, verrucous papules and nodules located on sun-exposed areas. HLE must be clinically and histologically differentiated from keratoacanthoma, hypertrophic lichen planus, squamous cell carcinoma and plaque type psoriasis. CD123 positivity and transepidermal elimination of elastin have recently been reported to distinguish HLE ^1, 2^.

## Report of case

**Figure 1.**
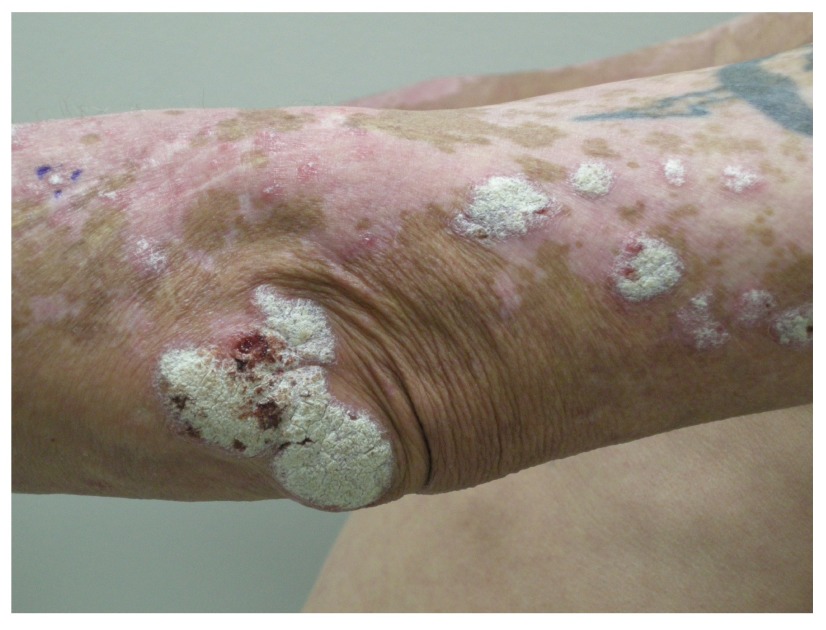
Clinical photo of hypertrophic lupus erythematosus. Hypertrophic lupus erythematosus presenting as a verrucous plaque on the patient’s elbow.

**Figure 2.**
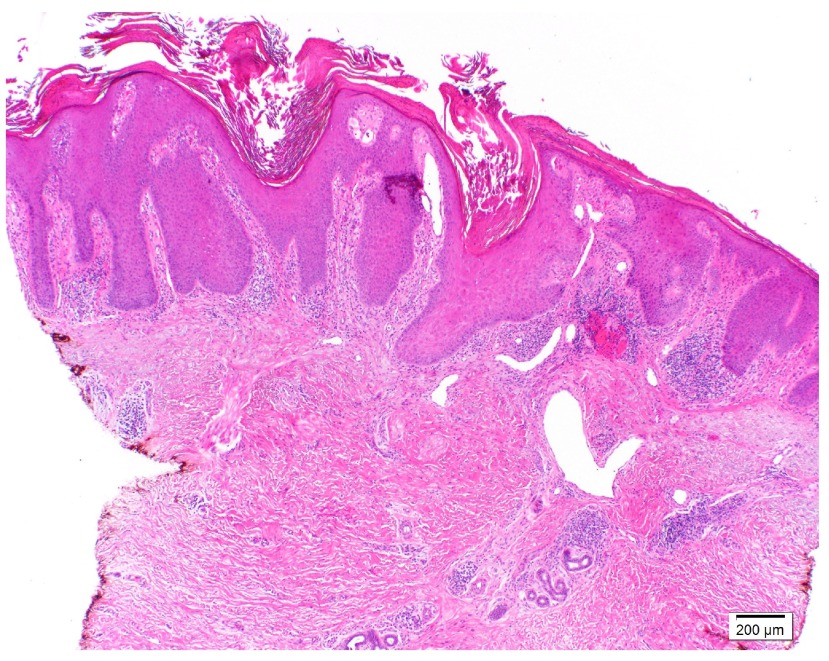
Histological photo of hypertrophic lupus erythematosus. Hypertrophic lupus erythematosus displays epidermal acanthosis and expansion of follicular ostia with a superficial and deep perivascular and periappendageal intradermal lymphocytic infiltrate (hematoxylin and eosin, 40× magnification).

A 49-year-old, unemployed, white male presented with a three-year history of an expanding “rash”. He reported no constitutional symptoms. He had previously been treated with oral prednisone and an unknown topical steroid without improvement and was off all medications at our initial visit. The patient had a past medical history of hepatitis C. He denied a family history of skin or autoimmune diseases. Laboratory work-up was significant for positive anti-nuclear antibodies and anti-Ro antibodies. Physical exam revealed multiple hyperkeratotic, verrucous papules and nodules with white, scaly, cribriform centers overlying patches of depigmentation, erythema and atrophy on his bilateral arms ( Figure 1) and anterior legs. His face and scalp had several atrophic, depigmented patches. Two punch biopsies were obtained from separate lesions. Histological sections demonstrated an interface inflammatory pattern with deep peri-vascular and peri-appendageal lymphocytic infiltrate and rare plasma cells ( Figure 2). A diagnosis of HLE was made. The patient was prescribed clobetasol ointment 0.05% twice daily. At the three month follow-up, there was improvement of the hypertrophic lesions. The patient was subsequently lost to follow-up.

## Discussion

HLE was first described by Bechet in 1940 ^3^. Clinical diagnosis can be challenging as HLE can mimic psoriasis or even squamous cell carcinoma. Uitto *et al.* described two histological patterns of HLE One resembled hypertrophic lichen planus, while the other was similar to keratoacanthoma ^4^. Daldon *et al.* found that transepidermal elimination of elastin was present in 14 cases of HLE ^1^. Recently, Ko *et al.* reported that a band of CD123 positive cells at the dermal-epidermal junction was characteristic of five cases of HLE ^2^.

**Figure 3.**
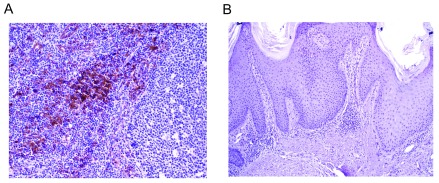
( **A**): Example of positive CD123 immunohistochemical stain from tonsil control tissue (200× magnification). ( **B**): CD123 immunohistochemical stain on the biopsy of hypertrophic lupus erythematosus from the reported patient. Only rare plasmacytoid dendritic cells are seen in the dermis. The stain is negative for the dense band of CD123 positive cells that Ko *et al.* described in hypertrophic lupus erythematosus (100× magnification).

In this patient, we examined these two recently described histologic features of HLE. Interestingly, both CD123 positivity and transepidermal elimination of elastin were not present in this case ( Figure 3). However, the histological and clinical findings were most consistent with HLE. The etiology of this discrepancy is unknown and further studies are needed to clarify the utility of CD123 positivity and transepidermal elimination of elastin in the diagnosis of hypertrophic lupus erythematosus.

There is no definitive treatment for HLE. Options include topical or intralesional steroids, hydroxychloroquine, topical calcineurin inhibitors, topical or oral retinoids, thalidomide and surgical excision ^5, 6^. Winchester *et al.* reported on the efficacy ustekinumab, an inhibitor of IL-12 and IL-23 ^7^.

This case highlights the discrepancies of CD 123 positivity and absence of transepidermal elimination of elastin in HLE.

## Consent

Written informed consent for publication of clinical details and clinical images was obtained from the patient.
