# Supplementary material for: Case Report: A case of hypertrophic lupus erythematosus with negative CD123 staining and absence of transepidermal elimination of elastin
Source: F1000Res. 2014 Jun 4;3:76. Originally published 2014 Mar 21. [Version 2] doi: 10.12688/f1000research.3267.2 (PMC4097349; doi:10.12688/f1000research.3267.2)
Supplement: Peer review history

# Peer review: Referee response for version 2

**DOI:** 10.5256/f1000research.4723.r5159

**Published:** 2014-06-18

**Authors:** Theresa Lu

**Affiliations:** 1 Autoimmunity and Inflammation Program and Pediatric Rheumatology, Hospital for Special Surgery, New York, NY, USA

The authors have now addressed all the concerns.

I have read this submission. I believe that I have an appropriate level of expertise to confirm that it is of an acceptable scientific standard.

# Peer review: Referee response for version 2

**DOI:** 10.5256/f1000research.4723.r5010

**Published:** 2014-06-17

**Authors:** Victoria Werth

**Affiliations:** 1 Department of Dermatology, University of Pennsylvania, Philadelphia, PA, USA

The authors successfully addressed the reviewer comments in this case report.

I have read this submission. I believe that I have an appropriate level of expertise to confirm that it is of an acceptable scientific standard.

# Peer review: Referee response for version 2

**DOI:** 10.5256/f1000research.4723.r5015

**Published:** 2014-06-05

**Authors:** Jordan Reynolds

**Affiliations:** 1 Department of Anatomic Pathology, Cleveland Clinic, Cleveland, OH, USA

This article is well written but needs a few additions to improve it:

1. Please elaborate on what CD 123 is, and how it is used for diagnosing HLE. It is not common knowledge and should be expanded upon.
2. It might also be worth commenting on this paper by Miyashita A *et al.* (2013, *Acta derm venereal*) in which the authors showed patients with CD123 positive cells responded better to treatment than patients with low CD123. Do you think any of this could be supported or refuted by your cases?

I have read this submission. I believe that I have an appropriate level of expertise to confirm that it is of an acceptable scientific standard.

# Peer review: Referee response for version 1

**DOI:** 10.5256/f1000research.3507.r4445

**Published:** 2014-04-29

**Authors:** Theresa Lu

**Affiliations:** 1 Autoimmunity and Inflammation Program and Pediatric Rheumatology, Hospital for Special Surgery, New York, NY, USA

This report describes a case of hypertrophic lupus erythematosus based on clinical and histopathologic criteria that is negative for CD123 and elastin elimination. Negative data is important. However, as the emphasis is on the lack of CD123 and the lack of transepidermal elastin elimination, it would be good to show the negative results. For the CD123 stain, it would be good to show a positive control to make sure that the antibody really worked.

I have read this submission. I believe that I have an appropriate level of expertise to confirm that it is of an acceptable scientific standard, however I have significant reservations, as outlined above.

# Peer review: Referee response for version 1

**DOI:** 10.5256/f1000research.3507.r4229

**Published:** 2014-04-14

**Authors:** Victoria Werth

**Affiliations:** 1 Department of Dermatology, University of Pennsylvania, Philadelphia, PA, USA

This is a case of hypertrophic lupus erythematosus that is described as unusual in pathologic presentation.

The title needs to indicate absence of transepidermal elimination of elastin. It is currently unclear if transepidermal elmination of elastin was present.

The order of treatment described for HLE is confusing.  One would start with hydroxychloroquine, then add quinacrine to hydroxychloroquine, with topicals as adjunctive therapy. Oral retinoids,  thalidomide, and immuosuppressives would be options. Given that frequently there are multiple lesions that may actually koebnerize in a surgical scar, one would not include surgical excision as an option.

The report cited in favor of TNF-alpha inhibitor is on ustekinumab, which is not a TNF inhibitor. This needs revision.

Information about the antibody used for CD123 staining, as well as whether frozen or fixed tissue was used, is important. Anti-CD123 staining is not as good on fixed tissue. Were there any positive controls stained simultaneously?

I have read this submission. I believe that I have an appropriate level of expertise to confirm that it is of an acceptable scientific standard, however I have significant reservations, as outlined above.
